# Whole-exome sequencing identified four loci influencing craniofacial morphology in northern Han Chinese

**DOI:** 10.1007/s00439-019-02008-6

**Published:** 2019-04-09

**Authors:** Wei Wu, Guiying Zhai, Zejun Xu, Bo Hou, Dahua Liu, Tianyi Liu, Wei Liu, Fu Ren

**Affiliations:** 1 School of Humanities and Management, Jinzhou Medical University, Jinzhou, 121001 Liaoning People’s Republic of China; 2 Biological Anthropology Institute, Jinzhou Medical University, No. 40, Section 3, Songpo Road, Linghe District, Jinzhou, 121001 Liaoning People’s Republic of China; 3 Liaoning Province Key Laboratory of Chinese Physical Characteristics Research (LPKL-CPCR), Jinzhou, 121001 Liaoning People’s Republic of China; 4 Department of Plastic and Maxillofacial Surgery, Uppsala University, Uppsala, Sweden

## Abstract

Facial shape differences are one of the most significant phenotypes in humans. It is affected largely by skull shape. However, research into the genetic basis of the craniofacial morphology has rarely been reported. The present study aimed to identify genetic variants influencing craniofacial morphology in northern Han Chinese through whole-exome sequencing (WES). Phenotypic data of the volunteers’ faces and skulls were obtained through three-dimensional CT scan of the skull. A total of 48 phenotypes (35 facial and 13 cranial phenotypes) were used for the bioinformatics analysis. Four genetic loci were identified affecting the craniofacial shapes. The four candidate genes are *RGPD3*, *IGSF3*, *SLC28A3,* and *USP40*. Four single-nucleotide polymorphism (SNP) site mutations in *RGPD3*, *IGSF3,* and *USP40* were significantly associated with the skull shape (*p* < 1×10^−6^), and three SNP site mutations in *RGPD3*, *IGSF3,* and *SLC28A3* were significantly associated with the facial shape (*p* < 1×10^−6^). The rs62152530 site mutation in the *RGPD3* gene may be closely associated with the nasal length, ear length, and alar width. The rs647711 site mutation in the *IGSF3* gene may be closely associated with the nasal length, mandibular width, and width between the mental foramina. The rs10868138 site mutation in the *SLC28A3* gene may be associated with the nasal length, alar width, width between tragus, and width between the mental foramina. The rs1048603 and rs838543 site mutations in the *USP40* gene may be closely associated with the pyriform aperture width. Our findings provide useful genetic information for the determination of face morphology.

## Introduction

Human facial shapes are species-specific and have individual specificity. The individual specificity of human facial shapes plays an important role in human personal identity and social activities (Koblenzer 2003; Zhang and Gao 2009). Facial features are affected by genetic environmental factors and their interactions. It has been shown that the heritability of most facial features exceeds 60% (Crouch et al. 2018; Djordjevic et al. 2016). The craniofacial shape has been found to be mainly determined by genetic factors (Relethford 1994; Relethford and Harpending 1994), and the shape of the skull determines the basic shape of the face (Lieberman et al. 2000). The correlation between the skull and facial shapes has also been used in forensic practice by forensic anthropologists for processes such as facial image imposition and craniofacial approximation (Claes et al. 2010; Yoshino et al. 1995). Research into the genetic basis of the skull shape is, therefore, crucial for further studies of the genetic variations of facial shapes. However, there is a lack of research on the molecular genetic basis of facial features.

To date, the published papers have shown whole-genome association analyses of human facial shapes (Adhikari et al. 2016; Cha et al. 2018; Claes et al. 2018; Cole et al. 2016; Lee et al. 2017; Liu et al. 2012; Paternoster et al. 2012; Qiao et al. 2018; Shaffer et al. 2016). Researchers have successfully applied these results to human facial modeling predictions and achieved satisfactory results (Claes et al. 2014). The subjects of the published studies were Europeans, Latin Americans, Africans, and Koreans. Whole-genome association analyses of facial morphology have shown association with *PAX3*, *PRDM16*, *TP63*, *C5orf50*, *COL17A1*, *DCHS2*, *RUNX2*, *GLI3*, *PAX1*, *EDAR*, *MAFB*, *PAX9*, *MIPOL1*, *ALX3*, *HDAC8*, *CACNA2D3*, *SCHIP1*, *PDE8A*, *OSR1*-*WDR35*, *HOXD1*-*MTX2*, *WDR27*, *SOX9, DHX35*, etc. However, the results were not consistent among the above-studied population (except PAX3, which was associated with facial morphology in a few of published papers), indicating that ethnic factors had a large impact on facial features.

Therefore, the present study aimed to identify genetic variants influencing craniofacial morphology in northern Han Chinese through whole-exome sequencing (WES) and Sanger Sequencing.

## Materials and methods

Study subjects 50 volunteers, including 13 males and 37 females, were recruited from three universities in the Jinzhou area, Liaoning, China. To minimize the impact of non-genetic confounding factors, such as region, ethnicity, age, and BMI, we strictly used the following criteria to select volunteers for this study: (1) no craniofacial surgery, trauma history and craniofacial deformity; (2) no genetic kinship each other; (3) normal Han adults who had been living in Liaoning for at least three generations and were between 20 and 30 years of age; and (4) normal body size with a BMI of 18.5–24. This study was approved by the Ethics Committee of Jinzhou Medical University. All participants provided written informed consent. Venous blood collection and a head CT scan were completed between March 2017 and July 2017 following the standard procedures.

Facial and skull phenotypic measurements using three-dimensional (3D) CT scanning of the head were used to collect craniofacial phenotype information and to reconstruct the 3D face and skull images based on the difference in gray values of the acquired CT data. The craniofacial landmarks on the CT were then marked by group discussion as previously described (Evison et al. 2010). The Euclidean distance between the landmarks was calculated to replace the directly measured distance, which avoided the inaccuracies of mapping, rotation, and other issues caused by selection of a coordinate system. The convenience and reliability of this morphometric method have been proven by numerous studies (Lele and Richtsmeier 1991, 1995; Subhash Lele 1993; Starbuck et al. 2015).

The VG Studio MAX 2.2 software (Volume Graphics Co., Ltd., Heidelberg, Germany) was used to perform 3D facial and skull reconstruction using CT images from the 50 subjects. This software was also used simultaneously to measure the 3D coordinates of the facial and skull landmarks.

**Fig. 1:**
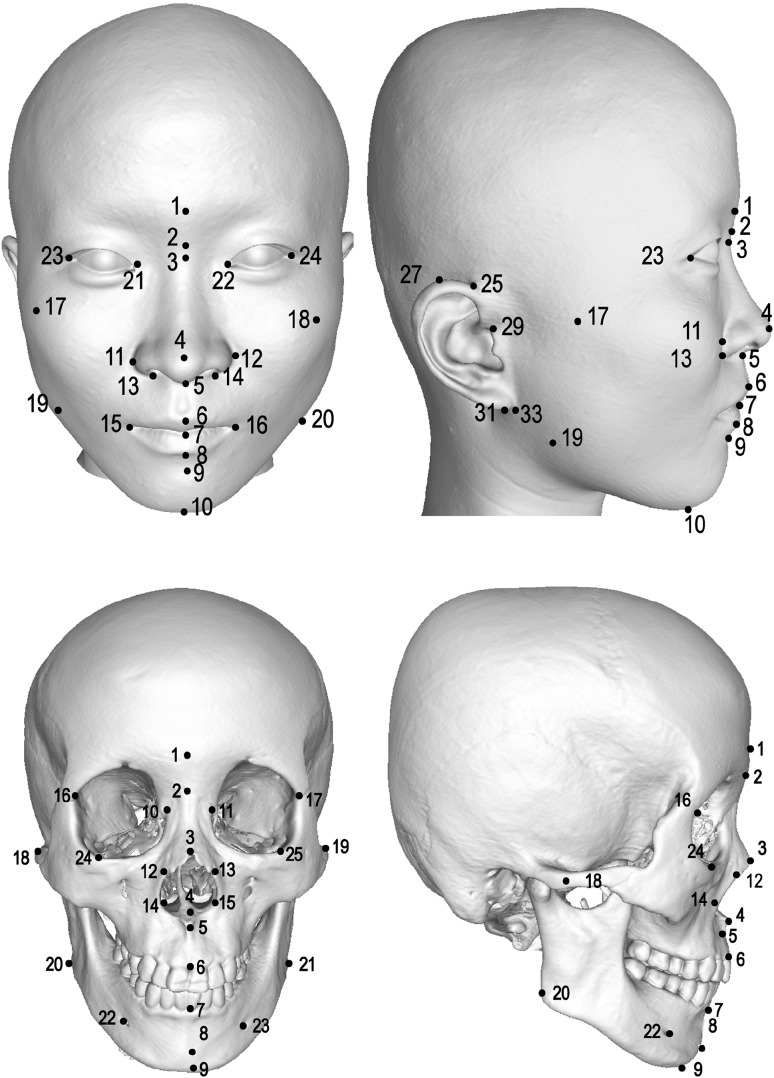
Facial and skull landmarks used in this study. Facial landmarks: 1 glabella (g); 2 nasion (n); 3 sellion (sl); 4 pronasale (prn); 5 subnasale (sn); 6 labrale superius (ls); 7 stomion (sto); 8 labrale inferius (li); 9 supramentale (sm); 10 gnathion (gn); 11, 12 alare (al); 13, 14 subalare (sal); 15, 16 cheilion (ch); 17, 18 zygion (zy); 19, 20 gonion (go); 21, 22 entocanthion (en); 23, 24 ectocanthion (ex); 25, 26 otobasion superius (obs); 27, 28 superaurale (sa), 29, 30 tragion (t); 31, 32 subaurale (sba); 33, 34 otobasion inferius (obi). Skull landmarks: 1 glabella (G); 2 nasion (N); 3 rhinion (RHI); 4 nasal spine (SP); 5 subspinale (SS); 6 exoprothion (EPR); 7 infradentale anterius (IDA); 8 pogonion (PG); 9 gnathion (GN); 10, 11 maxillofrontale (MF); 12, 13 naso-maxillary junction (NMJ); 14, 15 lateral piriform point (LP); 16, 17 frontomalare orbitale (FMO); 18, 19 zygion (ZY) 20, 21 gonion (GO); 22, 23 mentale (ML); 24, 25 orbitale (OR)

In this study, a total of 34 facial landmarks were measured (Ferrario et al. 1998), including 10 landmarks on the facial midline and 12 landmarks each on the left and right sides of the face. A total of 25 skull landmarks were measured (Adams et al. 2004; Kragskov et al. 1997; Moreddu et al. 2013), including 9 landmarks on the midline and 8 landmarks each on the left and right skull (Fig. 1). The WinEDMA 1.0.1 software (http://c.faculty.umkc.edu/colet) was used to calculate the Euclidean distances between landmarks. The above-mentioned reports (Adhikari et al. 2016; Cha et al. 2018; Claes et al. 2018; Cole et al. 2016; Lee et al. 2017; Liu et al. 2012; Paternoster et al. 2012; Qiao et al. 2018; Shaffer et al. 2016) concluded that gene loci of facial phenotypes were mostly associated with the shapes of the nose, mouth, and mandible, especially the nasal region. Therefore, this study selected phenotypes mostly from these regions. A total of 48 phenotypes were selected, including 35 facial phenotypes and 13 cranial phenotypes (the details are listed in Table 1).

**Table 1:** Craniofacial phenotypes used in this study

Name of phenotype	Description of phenotype
Facial phenotypes used in this study (35)	
n–prn	Euclidean distance from nasion to pronasale
sl–prn	Euclidean distance from sellion to pronasale
n–sn	Euclidean distance from nasion to subnasale
sl–sn	Euclidean distance from sellion to subnasale
prn–sn	Euclidean distance from pronasale to subnasale
sn–ls	Euclidean distance from subnasale to labrale superius
ls–sto	Euclidean distance from labrale superius to stomion
ls–li	Euclidean distance from labrale superius to labrale inferius
sto–li	Euclidean distance from stomion to labrale inferius
ch_r–ls	Euclidean distance from right cheilion to labrale superius
ch_r–li	Euclidean distance from right cheilion to labrale inferius
ch_l–ls	Euclidean distance from left cheilion to labrale superius
ch_r–li	Euclidean distance from right cheilion to labrale superius
ch_r–ch_l	Euclidean distance between the left and right cheilions
g–sm	Euclidean distance from glabella to supramentale
g–gn	Euclidean distance from glabella to gnathion
sm–gn	Euclidean distance from supramentale to gnathion
t_r–t_l	Euclidean distance between the left and right tragion
obs_r–obi_r	Euclidean distance from the right otobasion superius to the right otobasion inferius
obs_l–obi_l	Euclidean distance from the left otobasion superius to the left otobasion inferius
sa_r–sba_r	Euclidean distance from the right superaurale to the right subaurale
sa_l–sba_l	Euclidean distance from the left superaurale to the left subaurale
al_r–al_l	Euclidean distance between the right alare and the left alare
sal_r–al_r	Euclidean distance from the right subalare to the right alare
sal_r–al_l	Euclidean distance from the right subalare to the left alare
sal_l–al_r	Euclidean distance from the left subalare to the right alare
sal_l–al_l	Euclidean distance from the left subalare to the left alare
sal_l–sal_r	Euclidean distance from the left subalare to the right subalare
go_r–go_l	Euclidean distance between the right gonion and the left gonion
en_l–ex_l	Euclidean distance from the left entocanthion to the left ectocanthion
en_r–en_l	Euclidean distance between the right entocanthion and the left entocanthion
zg_r–zg_l	Euclidean distance between the right zygion and the left zygion
Morphological facial index	The quotient of the height and the width of the morphological surface is multiplied by 100
Zygomatic mandibular index	The width between the gonions is divided by the width of the surface and multiplied by 100
Nasal index	The nasal width is divided by the nasal height and multiplied by 100
Skull phenotypes used in this study (13)	
N–RHI	Euclidean distance from nasion to rhinion
RHI–SP	Euclidean distance from rhinion to spinale
RHI–SS	Euclidean distance from rhinion to subspinale
FMO_R–FMO_L	Euclidean distance between the right frontomalare and the left frontomalare
OR_R–OR_L	Euclidean distance between the right orbitale and the left orbitale
MF_R–FMO_R	Euclidean distance from the right maxillofrontale to the right frontomalare
MF_L–FMO_L	Euclidean distance from the left maxillofrontale to the left frontomalare
MF_R–MF_L	Euclidean distance from the right maxillofrontale to the left maxillofrontale
NMJ_R–NMJ_L	Euclidean distance between the right naso-maxillary junction and the left naso-maxillary junction
LP_R–LP_L	Euclidean distance between the right lateral piriform and the left lateral piriform
ZG_R–ZG_L	Euclidean distance between the right and the left zygion
GO_R–GO_L	Euclidean distance between the right gonion and the left gonion
ML_R–ML_L	Euclidean distance between the right mentale and the left mentale

### WES and whole-genome association analysis

WES and whole-genome association analysis were performed by Beijing Novogene Technology Co., Ltd. (Beijing, China, http://www.novogene.com). The quality of DNA samples was evaluated by agarose gel electrophoresis analysis. The DNA samples that were accurately quantified as greater than 0.6 µg by Qubit were used for library construction. The Agilent’s liquid chip capture system was applied to efficiently enrich the DNA containing all human exon regions. High-throughput deep sequencing was subsequently performed on the Illumina platform. The Agilent SureSelect Human All Exon V6 Kit was used for library construction and the capture experiments. After library construction, Qubit 2.0 was used for the preliminary quantification. The Agilent 2100 system was subsequently used to verify the library insert size. The Illumina platform was used for sequencing according to the effective concentration of the library and the data output requirements. High-throughput paired-end sequencing was performed (paired-end 150 bp, PE150).

After WES was completed, a bioinformatics analysis was performed that included three main aspects: sequencing data quality assessment, SNP detection, and whole-genome association analysis. The sequencing data quality control requirements were as follows: the sequencing error rate of each base position was less than 1%, the average Q20 ratio was greater than 90%, the average Q30 ratio was greater than 80%, the average error rate was less than 0.1%, the sequencing reads achieved an alignment rate of 95% or greater, and the read depth of the base at one position reached 10X or more.

The SNP testing was performed as follows: high-quality sequences were aligned with the human reference genome (GRCh37/hg19) to detect variant information in the sample, and the detected variations were analyzed and annotated.

### Sanger sequencing

Sanger sequencing validation in this study was performed by the BGI Beijing Branch (http://www.genomics.cn). Premier 3.0 was used for primer design; the primer sequences are listed in Table 5. The PCR products were purified using Millipore 96 purification plates. The sequencing reaction mixture was as follows: 2 μL of the buffer mix (Bigdye 3.1, 5× sequencing buffer in H_2_O, 2 μL of the purified PCR product, and 1 μL of the primer (5 mmol/L). The sequencing reaction program was as follows: 95 °C 15 s → (95 °C 15 s → 50 °C 5 s → 60 °C 90 s) × 35 cycles → stop reaction. Electrophoresis was used to detect the PCR-amplified products.

### Statistical analysis

The GEMMA (Genome-wide Efficient Mixed Model Association algorithm) software was used to construct a linear mixed model and calculate the association between the 48 phenotypes and genotypes (SNP variant loci) (Zhou and Stephens 2012). A mixed linear model was constructed using GEMMA software in R studio: *y* = *Wα* + *xβ* + *u* + *ε*, *y*: *n* quantitative traits of individuals; *W*: covariate (gender); *x*: *n* individual genotypes; *β*: slope coefficient, the size of the genotype. A mixed linear model can eliminate confounding results more accurately, and can directly reflect the relationship between craniofacial phenotype and genotype. Combining other studies of GWAS correlation analysis with our stringent requirements for quality control, our *p* value threshold is set to 1E−6 to avoid the effects of other mixing factors on experimental results. Therefore, the *p* values of less than 1 × 10^−6^ were regarded as statistically different.

## Results

### Craniofacial phenotypic measurement

Using the facial photographs of 50 individuals, we accessed 35 facial features and 13 cranial features. The craniofacial phenotypic measurements of adult northern Han Chinese are shown in Table 2.

**Table 2:** Craniofacial phenotypic measurements of adult northern Han Chinese

Name of phenotype	Mean (Euclidean distance)	SD
Facial phenotypes used in this study (35)		
n–prn	48.43	4.33
sl–prn	40.50	4.51
n–sn	58.84	4.45
sl–sn	50.43	4.57
prn–sn	19.54	2.42
sn–ls	12.89	1.90
ls–sto	10.00	2.09
ls–li	18.58	3.30
sto–li	9.92	1.73
ch_r–ls	30.05	2.79
ch_r–li	28.43	2.94
ch_l–ls	30.96	2.78
ch_r–li	29.25	3.04
ch_r–ch_l	49.22	4.45
g–sm	108.34	5.27
g–gn	138.23	8.53
sm–gn	33.16	6.26
t_r–t_l	151.85	8.59
obs_r–obi_r	51.85	4.55
obs_l–obi_l	50.27	3.92
sa_r–sba_r	61.30	3.95
sa_l–sba_l	60.45	3.89
al_r–al_l	133.77	7.83
sal_r–al_r	38.27	3.33
sal_r–al_l	9.96	2.29
sal_l–al_r	32.53	3.37
sal_l–al_l	33.32	3.99
sal_l–sal_r	8.97	2.27
go_r–go_l	26.16	5.15
en_l–ex_l	28.50	3.06
en_r–en_l	34.17	3.42
zg_r–zg_l	149.60	7.59
Morphological facial index	83.36	5.95
Zygomatic mandibular index	89.47	3.93
Nasal index	65.21	5.58
Skull phenotypes used in this study (13)		
N–RHI	25.95	3.07
RHI–SP	30.85	3.08
RHI–SS	34.76	2.76
FMO_R–FMO_L	96.10	4.05
OR_R–OR_L	75.63	5.63
MF_R–FMO_R	39.14	1.99
MF_L–FM0_L	38.73	2.07
MF_R–MF_L	20.96	3.35
NMJ_R–NMJ_L	18.11	2.53
LP_R–LP_L	27.24	2.13
ZG_R–ZG_L	132.66	6.13
GO_R–GO_L	97.84	6.20
ML_R–ML_L	48.89	2.86

### WES and whole-genome association analysis

**Fig. 2:**
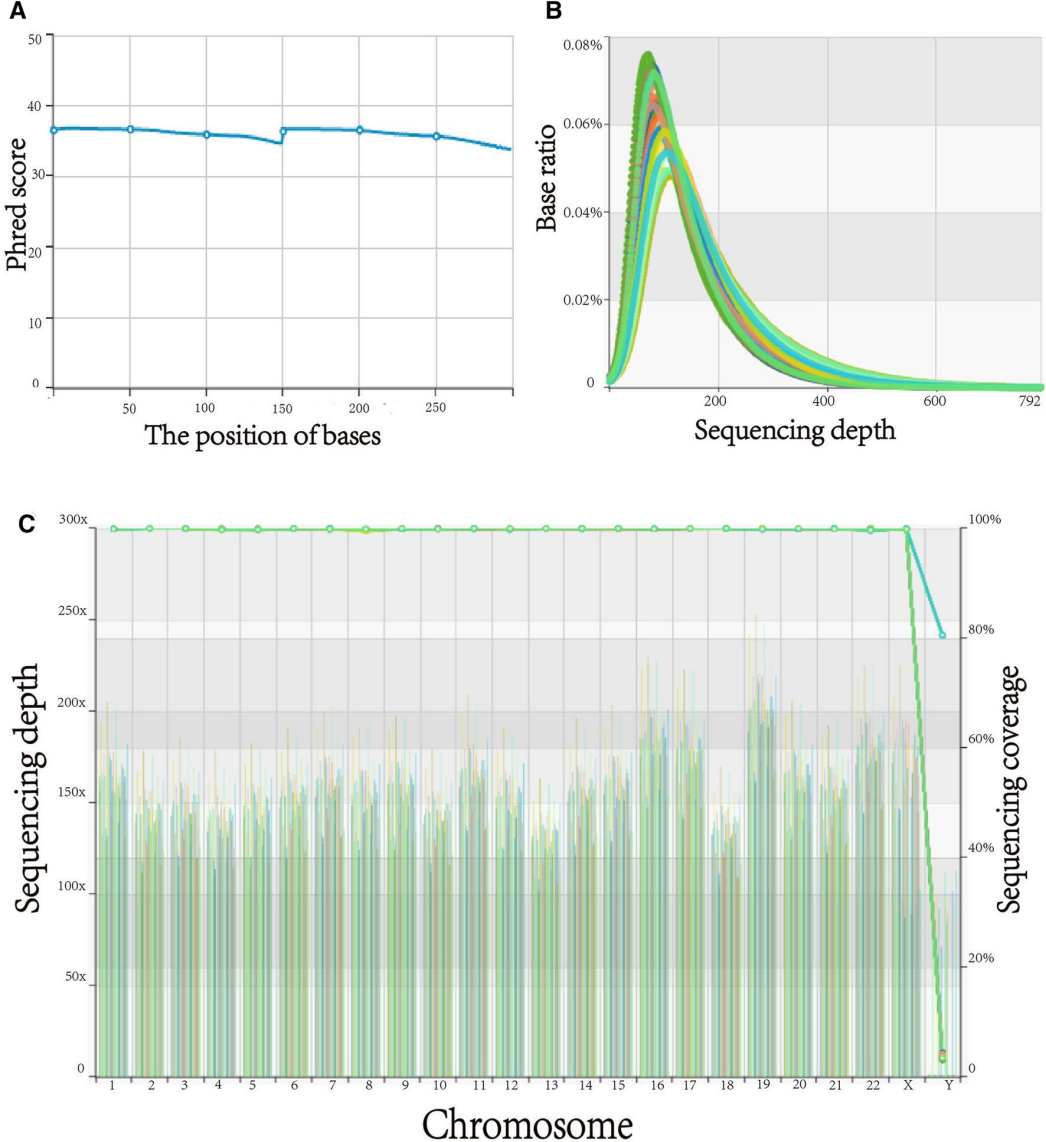
Error rate distribution along the reads. **a** The distribution of the average quality value (Phred score) of the WES data. **b** The depth of the WES; the abscissa indicates the depth of sequencing, and the ordinate indicates the ratio of bases with a sequencing depth of *X*. **c** The coverage depth and coverage rate of each chromosome; the abscissa indicates the chromosome number, the left ordinate indicates the average coverage depth, and the right ordinate indicates the coverage rate

**Fig. 3:**
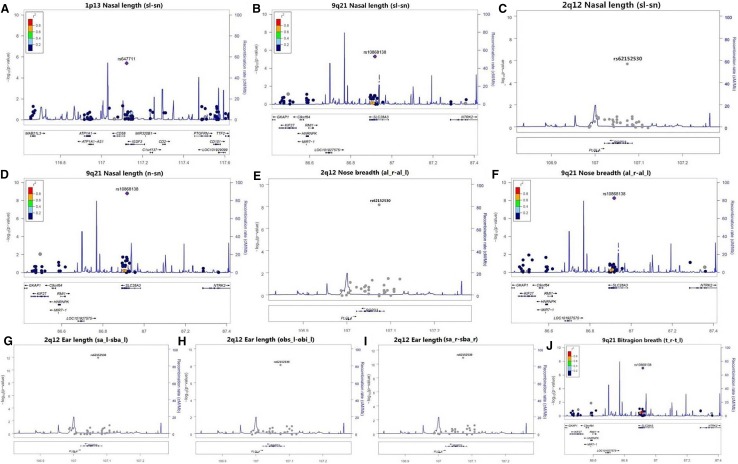
The whole-exome region showing genome-wide significant associations with facial traits. The SNP site mutations at 1p13 (rs647711), 9q21 (rs10868138), and 2q12 (rs62152530) have stronger associations with the facial morphology of the Han people of northern China. A G > C missense mutation occurred at rs647711 (*IGSF3*). This SNP site mutation is closely associated with one facial phenotype (nose length) (**a**). A T > C missense mutation occurred at rs10868138 (*SLC28A3*). This SNP site mutation is closely associated with four facial phenotypes (nose length, tragus width, nasal width n–sn, sl–sn, t_r–t_l, and al_r–al_l) (**b**, **d**, **f**, **j**). A C > T missense mutation occurred at rs62152530 (*RGPD3*). This SNP mutation is closely associated with four facial phenotypes (nose length, ear length, and nasal width) (**c**, **h**, **i**, **e**). Here, the LocusZoom plot used the linkage disequilibrium (LD) information in the hg19 1000 Genome Nov 2014 ASN (East Asia) database to obtain the *r*^2^. Since no LD information was available in the region of the database where the rs62152530 site was located, the image of this locus did not have *r*^2^ information

**Fig. 4:**
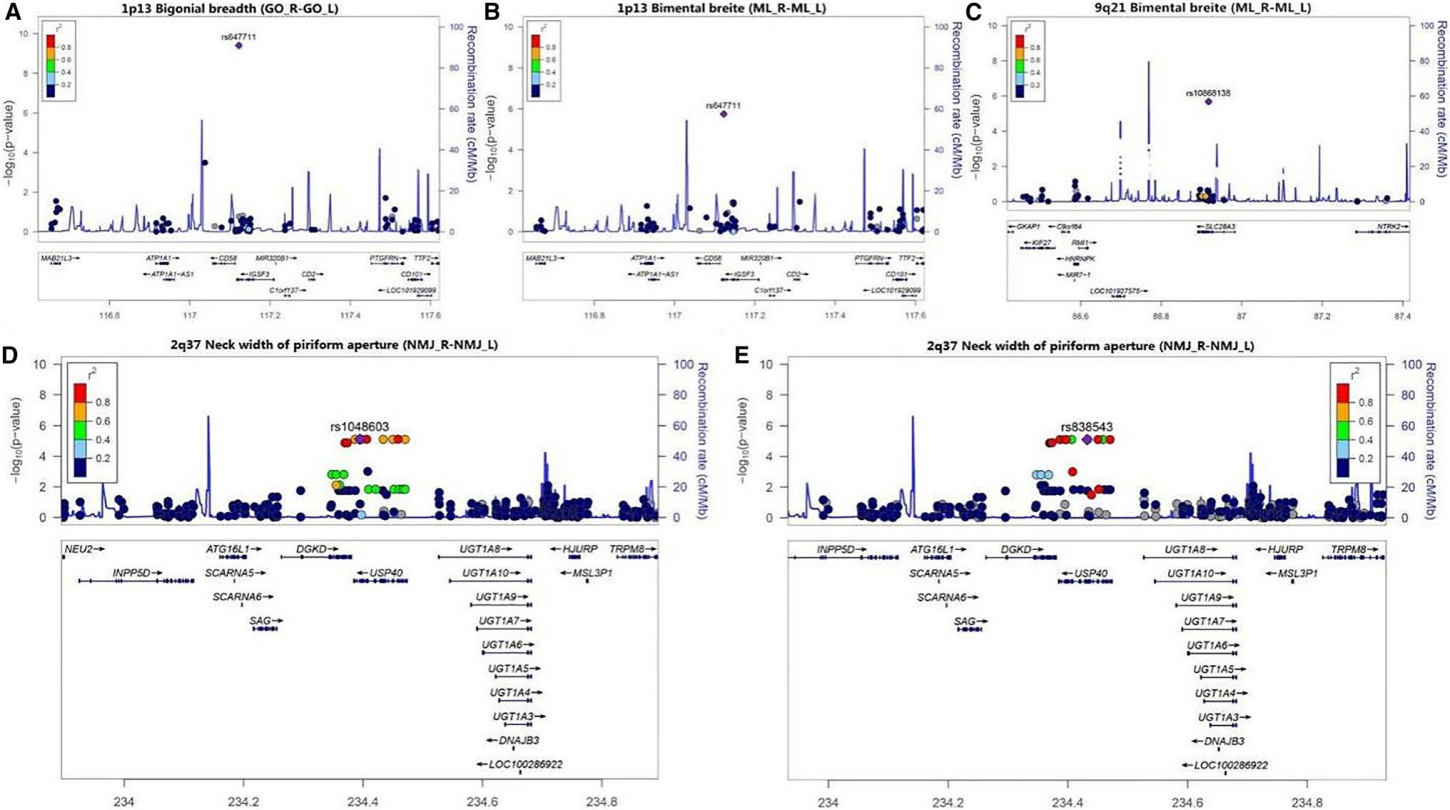
The whole-exome region showing genome-wide significant associations with the skull traits. A G > C missense mutation occurred at rs647711 (*IGSF3*). This SNP site mutation is closely associated with two skull phenotypes (width between the gonions and width between the mentales) (**a**, **b**). A T > C missense mutation occurred at rs10868138 *(SLC28A3*). This SNP site mutation is closely associated with one skull phenotype (width between the mentales) (**c**). Missense mutations of G > A occurred at both rs1048603 *(USP40*) and rs838543 (*USP40*). These two SNP site mutations are closely associated with one skull phenotype (pyriform aperture width) (**d**, **e**). Here, the LocusZoom plot was generated using the linkage disequilibrium (LD) information in the hg19 1000 Genome Nov 2014 ASN (East Asia) database

A total of 23,011 SNP sites were detected by WES. The sequencing quality control is shown in Table 3 and Fig. 2. The association analysis between the 23,011 SNP loci and 48 phenotypes showed that a C > T missense mutation occurred at rs62152530 (NM_001144013) in *RGPD3* (2q12). This SNP site mutation is closely associated with 4 facial phenotypes (sl–sn, obs_l–obi_l, sa_r–sba_r, sa_l–sba_l, and al_r–al_l). This mutation causes an Asp > Asn change in the protein amino acid sequence at position 111. A G > C missense mutation occurred at rs647711 (NM_001007237) in *IGSF3* (1p13). This SNP site mutation is closely associated with one facial phenotype (sl–sn) and two skull phenotypes (GO_R–GO_L and ML_R–ML_L), and causes an Asp > Glu change in the protein amino acid sequence at position 1020. A T > C missense mutation occurred at rs10868138 (NM_001199638) in *SLC28A3* (9q21). This SNP site mutation is closely associated with 4 facial phenotypes (n–sn, sl–sn, t_r–t_l, and al_r–al_l) and one skull phenotype (ML_R–ML_L). This mutation causes a Tyr > Cys change in the protein amino acid sequence at position 113. G > A missense mutations occurred at the rs1048603 (NM_018218) and rs838543 (NM_018218) sites in *USP40* (2q37). These two SNP site mutations are closely associated with one skull phenotype (NMJ_R–NMJ_L). The mutations caused a Cys > Thr change in the protein amino acid sequence at position 1123 and a Thr > Cys change at position 2033 (Table 4, Figs. 3, 4).

**Table 3:** Statistical analysis of the WES quality control data

Sample	Average data volume (*G*)	Average Q30 (%)	Average sequencing depth in bed region (*x*)	Alignment (%)	> 20*x* (%)	> 10*x* (%)
50 cases	7.6	95.25	158.51	99.85	99.04	99.51

**Table 4:** Craniofacial phenotype-related SNPs in northern Han Chinese

Associated trait	SNP	Gene	Chr	POS	Alleles	Beta	*p* value
Facial trait (7)							
Nasal length							
sl–sn	rs62152530	RGPD3	2q12	107,073,501	C > T	2.58	1.58 × 10^−6^
	rs647711	IGSF3	1p13	117,122,288	G > C	− 2.45	3.95 × 10^−6^
	rs10868138	SLC28A3	9q21	86,917,301	T > C	− 1.26	4.88 × 10^−6^
n–sn	rs10868138	SLC28A3	9q21	86,917,301	T > C	1.78	1.58 × 10^−9^
Nose breadth							
(al_r–al_l)	rs10868138	SLC28A3	9q21	86,917,301	T > C	− 1.27	5.42 × 10^−9^
Ear length							
obs_l–obi_l	rs62152530	RGPD3	2q12	107,073,501	C > T	2.86	6.29 × 10^−9^
sa_r–sba_r	rs62152530	RGPD3	2q12	107,073,501	C > T	3.60	9.23 × 10^−12^
sa_l–sba_l	rs62152530	RGPD3	2q12	107,073,501	C > T	3.85	8.51 × 10^−13^
Bitragion breadth							
(t_r–t_l) Skull trait(3)	rs10868138	SLC28A3	9q21	86,917,301	T > C	2.89	9.66 × 10^−8^
Bigonial breadth							
(GO_R–GO_L)	rs647711	IGSF3	1p13	117,122,288	G > C	4.97	3.91 × 10^−10^
Bimental breite							
(ML_R–ML_L)	rs647711	IGSF3	1p13	117,122,288	G > C	1.61	1.79 × 10^−6^
	rs10868138	SLC28A3	9q21	86,917,301	T > C	0.83	2.08 × 10^−6^
Neck width of piriform aperture							
(NMJ_R–NMJ_L)	rs1048603	USP40	2q37	234,394,487	G > A	3.55	7.71 × 10^−6^
	rs838543	USP40	2q37	234,432,017	G > A	3.67	7.82 × 10^−6^

### Sanger sequencing

**Fig. 5:**
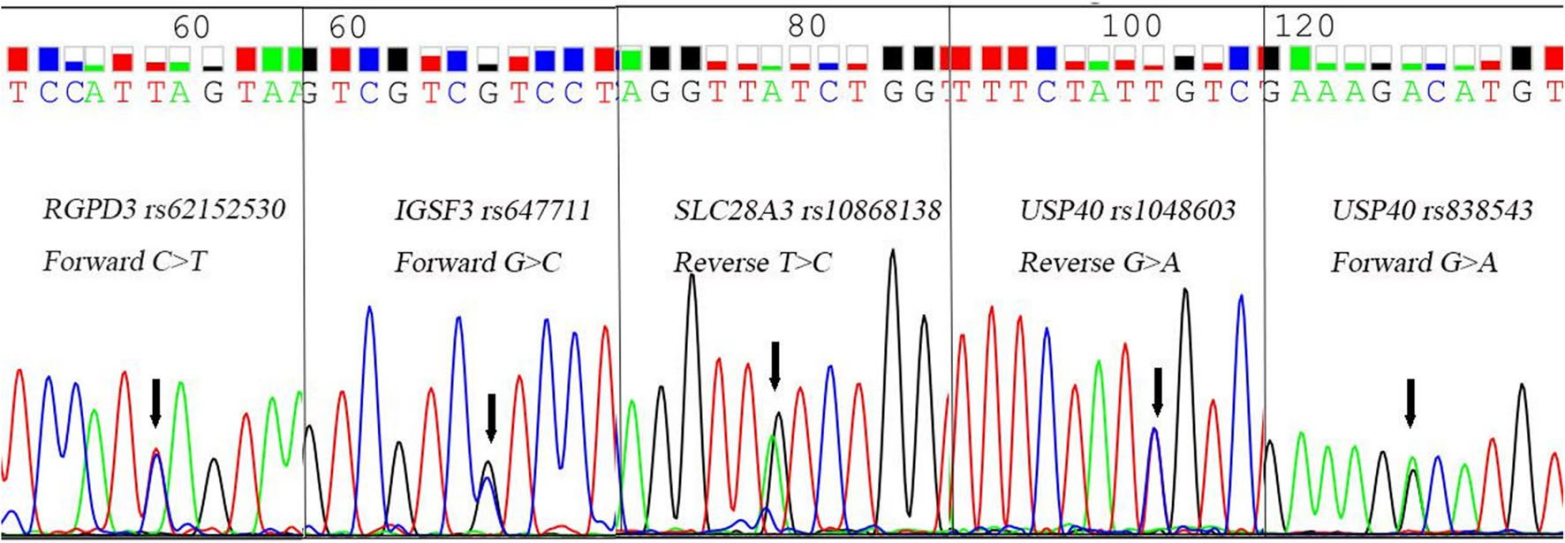
Sanger sequencing validated the mutations at the five SNP sites. A C > T missense mutation occurred at rs62152530 (*RGPD3*), a G > C missense mutation occurred at rs647711 (*IGSF3*), a T > C missense mutation occurred at rs10868138 (*SLC28A3*), and G > A missense mutations occurred at both rs1048603 (*USP40*) and rs838543 (*USP40*). The Sanger sequencing results confirmed that the five SNP sites identified by WES, indeed, had mutations

Sanger sequencing validation showed that rs62152530, rs647711, rs10868138, rs1048603, and rs838543 all had mutations that were consistent with the WES results. The mutation rates of these sites were 100% (50/50), 24% (12/50), 14% (7/50), 22% (11/50), and 20% (10/50), respectively. All of the sites were heterozygous mutations (Fig. 5).

## Discussion

We identified the SNP loci of four genes which were significantly associated with the craniofacial shape of northern Han Chinese. This result is the first report on the genetics of northern Han Chinese craniofacial shapes, which has significance for understanding craniofacial growth and development and improving the accuracy of human facial modeling based on DNA. Our findings could not replicate the previously published results (Table 5).

**Table 5:** Primer sequences used for PCR resequencing of the five SNP sites

Gene	SNP	Chr	POS	Primer sequence	Sequencing direction
*RGPD3*	rs62152530	2q12	107,073,501	ACTAAACTGTAAAATCCCTA	Forward
*IGSF3*	rs647711	1p13	117,122,288	CCCTCCCAAGGACTGCC	Forward
*SLC28A3*	rs10868138	9q21	86,917,301	TGATATTAAACCTCCCCTCA	Reverse
*USP40*	rs1048603	2q37	234,394,487	ACGTGCTGCTGAGGACAC	Reverse
*USP40*	rs838543	2q37	234,432,017	AGCCCTTGCTCCCTGAACG	Forward

Five SNP sites at 2q12, 1p13, 9q21, and 2q37 were found to be associated with craniofacial phenotypes in northern Han Chinese. These SNP sites are located in the four candidate genes *RGPD3* (RANBP2-Like and GRIP Domain Containing 3), *IGSF3* (Immunoglobulin Superfamily Member 3), *SLC28A3* (Solute Carrier Family 28 Member 3), and *USP40* (Ubiquitin Specific Peptidase 40).

*RGPD3* (Ciccarelli et al. 2005) is located in the Ran-binding protein-related gene cluster on chromosome 2 (cytogenetic location: 2q12.2; genomic coordinates (GRCh37/hg19) 107,021,136–107,084,832 and (GRCh38/hg38) 106,391,290–106,468,376). This gene is a protein-coding gene, and one of its related pathways is RNA transport. A study of new complex syndromes caused by the absence of 2q12.1–2q14.2 suggests that its absence may lead to a deformed appearance in child patients, which is manifested as a flat arcus supraorbitalis, wide alveolar ridge, high forehead, low ear, and other facial features (Stoeva et al. 2011). Because the *RGPD3* gene is located within the range of 2q12.1–2q14.2, *RGPD*3 is highly likely to be associated with these special facial features. In this study, we found that a C > T missense mutation occurred at rs62152530. This SNP site mutation is closely associated with the facial nasal length (sl–sn), ear length (obs_l–obi_l, sa_r–sba_r, and sa_l–sba_l), and nasal width (al_r–al_l), which partially overlap with the special facial features caused by the loss of 2q12.1–2q14.2, suggesting that *RGPD3* is associated with the morphology of the nose and ears.

*IGSF3* is located on chromosome 1 (cytogenetic location: 1p13.1; genomic coordinates (*GRCh38*): 1: 116,574,397–116,667,754) and is a member of the immunoglobulin superfamily. However, studies have not detected the *IGSF3* mRNA in peripheral blood leukocytes, the spleen, or the liver, suggesting that *IGSF3* is not involved in immune functions (Saupe et al. 1998). Foster et al. revealed that loss of *IGSF3* was associated with congenital nasolacrimal duct obstruction. RT-PCR analysis confirmed the presence of *IGSF3* transcripts in the embryonic mouse lacrimal gland, and *IGSF3* transcripts were detected in the lacrimal canaliculi and lacrimal glands 1 day after mice were born. Congenital lacrimal duct obstruction includes Hasner valve obstruction and congenital lacrimal duct atresia (including bony nasolacrimal duct atresia caused by bone malformation) (Foster et al. 2014). This finding suggests that *IGSF3* may be involved in the regulation of craniofacial developmental. In this study, a G > C missense mutation was found at rs647711 in *IGSF3*, and this SNP mutation was closely associated with the facial nasal length (sl–sn), the width between the gonions, and the width between the mentales (GO_R–GO_L and ML_R–ML_L) of the skull. This result suggests that *IGSF3* may not only participate in regulating development of the lacrimal ducts, but may also participate in the growth and development of other structures of the face and skull.

*SLC28A3,* also known as *CNT3* (Ritzel et al. 2001), is a solute carrier family member located on chromosome 9 [cytogenetic location: 9q21.32–q21.33; genomic coordinates (GRCh38): 9: 84,273,122–84,368,633], and is involved in the regulation of various cellular processes, including neurotransmission, vascular tone, nucleoside transport and metabolism, and other processes. *SLC28A3* has extensive specificity for the transport of pyrimidine and purine nucleosides (Barber et al. 2010). Studies have shown that *SLC28A3* is associated with obesity, and it may be associated with the lipid-lowering response of statins (Comuzzie et al. 2012; Guallar et al. 2007). Body fat content has a great influence on facial morphological characteristics, and thus, *SLC28A3* may have a certain correlation with facial morphology. In this study, a T > C missense mutation was found at rs10868138 in SLC28A3. This SNP site mutation is closely associated with the facial nasal length, the width between the tragus, the alar width (n–sn, sl–sn, t_r–t_l, and al_r–al_l), and the width between the mental foramina (ML_R–ML_L).

*USP40* is located on chromosome 2 [cytogenetic location: 2q37.1; genomic coordinates (GRCh38): 2:233, 475,518–233,566,788] and belongs to a large family of cysteine proteases. *USP40* modifies cellular proteins through ubiquitin by playing the role of a deubiquitinating enzyme (Quesada et al. 2004). USP40 is expressed in many organ tissues, and its biological functions, protein localization, and structural characterization are completely unknown. A few reports have linked this gene to Parkinson’s disease or glomerular function (Li et al. 2006; Takagi et al. 2017; Zhao et al. 2012). In this study, we found G > A missense mutations at rs1048603 and rs838543 in the *USP40* gene; these two SNP site mutations were closely associated with the width of the pyriform aperture. This result suggests that *USP40* has a wide range of biological functions and may participate in the regulation of craniofacial development.

Candidate genes for human facial variation and GWAS studies showed a few consistent results. Most of the novel genes identified in each study were different. In fact, racial factors are a major cause because the results come from different populations, such as Europeans, Americans, Africans and East Asians, etc. (Adhikari et al. 2016; Cha et al. 2018; Claes et al. 2018; Cole et al. 2016; Lee et al. 2017; Liu et al. 2012; Paternoster et al. 2012; Qiao et al. 2018; Shaffer et al. 2016). Even in the same race, such as in Europeans, few of the genes or loci identified in each study have been replicated (Adhikari et al. 2016; Lee et al. 2017; Paternoster et al. 2012). In addition, during the review process of this paper, Qiao et al. (2018) reported that, in the GWAS study of the discovery panel of Uyghurs, six significant loci were identified, four of which, rs1868752, rs118078182, s60159418 at or near UBASH3B, COL23A1, PCDH7, and rs17868256 were replicated in independent cohorts of Uyghurs or Southern Han Chinese, which are not insistent with our findings. In addition, the environment may be the likely cause of this situation, for example, even if the study participants in each study are Europeans, they are from different countries or from European descent/Americans. The inconsistency between Qiao’s and our findings may also be due to ethnic and/or environmental factors in the Uighur or South Han and the Northern Han Chinese. Detection of effective genes or loci affecting craniofacial morphology cannot ignore sample size. The authors acknowledge the limitation of a small sample size; only 50 subjects were selected due to funding limitation. However, the findings through WES and whole-genome association analysis in the Han Chinese are novel and significant in the understanding of the genetic basis of facial development.

Another possibility is that the measurement methods and phenotypic descriptions in each study are different. For phenotypic and genomic association analysis, the accuracy of phenotypic measurement is crucial. In the previous studies, 2D method was often used to measure craniofacial morphology. In the 2D method, even with 3D reconstructions, the linear distance between the two projection points is measured on a plane parallel to the coronal or sagittal plane when measuring the distance between two landmarks. However, the phenotypic accuracy of 2D image measurement is limited. Liu et al. (2012) indicated that 2D image noise, image size, and pixel resolution had an impact on phenotypic measurements, and some marker point coordinates cannot be measured on a 2D image. Cha et al. (2018) also showed that the main limitation of their research was that facial measurements were derived from 2D images rather than 3D images (they subsequently measured more facial features such as angle, ratio, curvature, width, height, etc.). In the present study, we used geometric morphometrics to extract the 3D coordinates of two landmarks and calculated the Euclidean distance between the two points. We believe that this new method improves the accuracy of our study. In addition, to minimize the measurement error, in this study, the two authors performed three geometric measurements on all coordinate points, respectively, using the average of the measured data as the final ones.

Overall, the present study not only identified four loci influencing craniofacial morphology in northern Han Chinese, but also established an approach for analyzing the genetic basis of human facial traits through WES combined with 3D CT scanning. Compared with GWAS, WES display is more accurate and cost-effective, and the sequencing depth can reach 120*x* and more, which can meet the research of large target and multi-target area. In this study, WES can more directly reflect the effect of protein-coding regions on craniofacial phenotypes.

Taken together, our results show that *RGPD3*, *IGSF3*, *SLC28A3*, and *USP40* have significant associations with the facial morphology of the northern Han people and that the associated phenotypes are concentrated in the nose and mandible regions. Although not the same genes as reported, it is consistent with the relevant regions. Of these genes, both *IGSF3* and *SLC28A3* are significantly associated with facial and cranial morphology, suggesting that genes that affect cranial morphology likely affect facial morphology, as well. Cranial morphology, in turn, should be incorporated into genomics research of facial morphology.
